# AI safety evaluation in an underrepresented population: real-world performance of clinical decision support and frontier language models on Medicaid patient messaging triage

**DOI:** 10.1186/s12911-026-03763-z

**Published:** 2026-08-18

**Authors:** Sanjay Basu, Sadiq Y. Patel, Parth Sheth, Bernardo Arevalo, Jeremy Schifberg, John Morgan, Rajaie Batniji

**Affiliations:** 1 University of California, San Francisco, San Francisco, CA USA; 2 Waymark, San Francisco, CA USA; 3 University of Pennsylvania, Philadelphia, PA USA; 4 Virginia Commonwealth University, Richmond, VA USA; 5 University of California, San Francisco and Waymark, 2021 Fillmore St, 94115 San Francisco, CA USA

**Keywords:** Clinical decision support, Patient safety, Large language models, Primary care, Medicaid, Text messaging triage, Reinforcement learning, Retrieval-augmented generation, Federally qualified health centers, Deployment validation

## Abstract

**Background:**

Studies of artificial intelligence tools used in patient triage have largely involved academic medical center cohorts, scripted patient-actor scenarios, or knowledge benchmarks. Populations that may rely on such tools due to constrained access to in-person care, including Medicaid patients, have been less fully evaluated.

**Objective:**

To compare combinations of safety guardrails added to artificial intelligence tools for triage of patient-initiated text messages in a multi-state Medicaid population.

**Methods:**

Retrospective evaluation of 2000 messages from Medicaid patients across three U.S. states (Virginia, Washington, Ohio) during January 2023 through November 2025. Three physicians independently adjudicated each message under blinded review; disagreements were resolved by majority and a senior-physician arbiter. Tools included a deployed decision support system with a Conservative Q-Learning controller, supervised baselines (XGBoost+sentence-BERT, logistic regression, rule-based guardrails), and frontier large language models (Claude Opus 4.7, GPT-5.5, Gemini 3.1 Pro, each with and without retrieval-augmented generation). Combinations spanned single tools, ensembles, cascades, multi-model consensus rules, and four-component guardrail compositions identified by a structured literature review. Thresholds and Platt calibration were fit on a held-out validation split and frozen before the test pass. Two pre-specified targets: an autonomous (physician-unassisted) triage benchmark of sensitivity and specificity both 0.80 or higher; and a sensitivity-floor target of 0.80 to 0.95 with clinician review of flagged messages.

**Results:**

Real-world messages had lower reading level (grade 4.6 versus 5.8) and more colloquialisms (59.1% versus 19.5%) than physician-scripted scenarios. Hazard prevalence on blinded physician review was 8.2% (165 of 2000). Two configurations met the sensitivity-floor target: a high-recall first-stage screen (sensitivity 0.855; 12.0 missed hazards and 729 alerts per 1,000 messages) and a disagreement-stratified clinician-review workflow (sensitivity 0.939; 5.0 missed hazards and 878 alerts per 1,000 messages). No configuration met the autonomous benchmark; sensitivity and specificity reached 0.594 and 0.592 for the best balanced single tool, 0.685 and 0.575 for the best balanced ensemble, and 0.297 and 0.874 for the highest-specificity cascade.

**Conclusions:**

No evaluated tool or combination was sufficiently accurate to enable physician-unassisted triage in this setting. Two configurations met the pre-specified sensitivity-floor target under clinician review of flagged messages, following the classical clinical-screening pattern.

**Supplementary Information:**

The online version contains supplementary material available at https://doi.org/10.1186/s12911-026-03763-z.

## Introduction

Patients now contact their primary care teams by written message in addition to visits and phone calls. The volume of messages sent through patient portals and through secure text-message lines to primary care teams rose during and after the 2020 to 2021 expansion of remote care [1]. Primary care appointments have at the same time become harder to schedule, and the wait is longer in lower-income neighborhoods [2]. More clinical questions therefore reach the practice as a written message rather than as a visit or call. Messages are typically reviewed first by medical assistants or care coordinators before being routed to a physician [3]. The physician inbox already holds laboratory results, refill requests, and system notifications, and the added message volume is associated with reductions in physician well-being [4]. The backlog concentrates at federally qualified health centers and other safety-net practices, which serve counties with documented primary care provider shortages [5]. Support staff who perform initial message triage are frequently in short supply at these practices. Artificial intelligence tools that scan incoming messages and flag clinical emergencies or urgent situations are of growing interest as a way to surface time-sensitive cases to clinicians without adding triage staff.

Artificial intelligence adapted to clinical triage has accelerated in scope and capability. The Articulate Medical Intelligence Explorer (AMIE) program evaluated a frontier large language model on synchronous text-chat consultations with standardized patient-actors [6], on accurate differential diagnosis from New England Journal of Medicine clinicopathologic case reports [7], and most recently in a 2026 multimodal demonstration on 105 scripted patient-actor scenarios across 32 non-multimodal and 9 multimodal qualitative axes rated by 18 specialists [8]. The MedHELM benchmark suite [9] catalogued frontier-model performance on knowledge-intensive medical tasks. Brodeur and colleagues evaluated a frontier language model on the reasoning tasks of an emergency-department physician using raw electronic health record text [10], and Ramaswamy and colleagues stress-tested a consumer-facing triage chatbot on 60 clinician-authored vignettes across 16 factorial conditions, observing 52% under-triage on clinical emergencies [11]. Recent patient-portal-message triage work by Liu and colleagues evaluated retrieval-augmented large language models on academic medical center portal correspondence at a single quaternary-care institution [12]. A natural next extension is evaluation in populations that may increasingly rely on artificial intelligence-assisted triage because traditional in-person care access is constrained, that communicate in colloquial or abbreviated text, that carry a higher comorbidity and behavioral-health burden, and that have higher baseline rates of medical mistrust and healthcare-interaction stigma [13] than the cohorts studied to date. To the authors’ knowledge, evaluation on Medicaid patient asynchronous text-based messaging (whether sent by short-message service to a secure line or as a patient-portal message) at scale has not been reported. Safety characteristics established on academic medical center cohorts and physician-scripted scenarios cannot be assumed to transfer to populations with these distinct linguistic and clinical profiles.

Patient-triage systems described in the published literature use different combinations of artificial intelligence tools: rule-based guardrails, supervised classifiers on bag-of-words or sentence-embedding features, reinforcement-learning controllers over hazard-category probability vectors with asymmetric reward structures, and prompted frontier large language models with safety-augmented prompts and optional retrieval-augmented generation [14]. More recent proposals add multi-model consensus rules, threshold-optimized cascades, and multi-component guardrail compositions drawn from current health artificial intelligence assurance frameworks. The relative safety performance of these tools and combinations on a Medicaid asynchronous text-messaging triage population has not been characterized head-to-head.

This study compares combinations of safety guardrails added to widely-used artificial intelligence tools for triage of patient-initiated text messages in a multi-state Medicaid managed care population. Two contributions are pursued. First, we characterize a previously underrepresented population (Medicaid patients across three U.S. states whose linguistic patterns differ from academic medical center cohorts and physician-scripted scenarios) and report the per-tool safety performance observed on this population. Second, we report the head-to-head performance of three concurrent-generation frontier large language models (Claude Opus 4.7, GPT-5.5, and Gemini 3.1 Pro Preview), configured with safety-augmented system prompts, against the deployed decision support architecture and classical supervised baselines, on the same patient messages and against the same blinded-physician-adjudicated reference labels.

## Methods

### Study design and population

This was a retrospective evaluation on text messages from 1679 Medicaid managed care patients enrolled in a multi-state clinically integrated network operating across three United States (US) states (Virginia, Washington, and Ohio), submitted between January 2023 through November 2025. 2000 messages comprised the held-out test set (Table 1); 1280 messages constituted the development set, which was further partitioned into a 570-message training subset for model fitting and a 710-message validation subset for threshold selection and Platt calibration. The test set, the training subset, and the validation subset were drawn from distinct messages. Patient-level overlap between training and test sets was 41 of 1,679 unique test-set patients (2.4%), reported transparently with a patient-disjoint sensitivity analysis in the Additional file 1.

**Table 1:** Population characteristics of the held-out real-world test set and the physician-scripted comparison scenarios

Characteristic	Real-world Medicaid test set	Physician-scripted comparison
Sample size, N	2000	41
Hazards adjudicated, n (%)	165 (8.25%)	27 (65.9%)
Benigns adjudicated, n (%)	1835 (91.75%)	14 (34.1%)
Reading level (Flesch-Kincaid grade), mean	4.6	5.8
Colloquialisms, % messages	59.1	19.5
Abbreviations, % messages	24.5	36.6
Implicit contextual references, % messages	7.8	29.3
Word count, mean	28.4	38.6

Caption: Sample sizes, hazard prevalence, and message-level linguistic feature distributions for the 2000-message real-world test set and the 41-case physician-scripted comparison set. Linguistic features were computed from the message text by the automated pipeline described in Methods and characterize the input presented to each tool, not any attribute of the patient. Patient-level characteristics are reported separately in Table 2. Reading level is the Flesch-Kincaid grade

Patient eligibility criteria were: enrollment in the participating Medicaid managed care entity in Virginia, Washington, or Ohio; and at least one text message sent to the clinical network during the study period. Eligibility was based on active enrollment rather than on any specific office visit, and no minimum or maximum interval between a message and an office visit was imposed; messages therefore arose in the context of ongoing managed care enrollment. No age restriction was applied. Messaging access is registered to the enrolled patient and identity verification is performed before each use; family members and caregivers are not issued separate messaging accounts. The exception is pediatric members, for whom a parent or guardian communicates on the member’s behalf under the member’s verified account: 136 (8.1%) of patients in the sample were younger than 18 years.

### Message curation and inclusion criteria

Messages were drawn from the full corpus of patient-initiated text correspondence to the clinical network during the study period. Messages were not short single-word greetings (“hi”, “hello”) but full patient inquiries comparable in length and content to patient-portal messages or short clinical emails (median length 38 words, interquartile range 18–72 words). Messages were screened to exclude administrative-only content (appointment scheduling, billing-only, prescription-refill-only) and to retain messages containing at least one clinical complaint, symptom description, or care-coordination request. The screening protocol is detailed in Additional file 1.

### Reference labeling and adjudication

Each message was independently reviewed by three physicians from the participating multi-state clinically integrated network. Reviewers were blinded to all artificial intelligence predictions throughout adjudication, and to one another’s labels during the initial review pass. For each message, each reviewer labeled (a) hazard presence (binary), (b) hazard category (from a pre-specified list of clinical categories spanning behavioral-health, substance-use, obstetric, pediatric, cardiac, neurological, metabolic, and environmental safety hazards), and (c) recommended triage action on the 8-point ordinal action scale (Action 1 = self-care; Action 8 = emergency services). Disagreements were resolved in two stages: first by majority vote (two of three reviewers agreeing constituted the reference label), and second by senior-physician arbiter review for residual splits. The two-stage majority-then-arbiter design was selected over a forced-unanimity design to preserve labels on genuinely ambiguous cases that nevertheless have a defensible reference action. The arbiter-resolved subset is identified in the released per-message prediction file for any reviewer who wishes to conduct a sensitivity analysis restricted to unanimously-labeled cases. Initial inter-rater agreement across the three independent reviewers (before majority and arbiter resolution) was substantial: Fleiss kappa was 0.82 for hazard presence (the primary endpoint) and 0.76 for hazard category. The complete labeling protocol is reported in Additional file 1.

### Demographics and equity

Patient characteristics were obtained by linking the frozen test set to the member record: each message identifier resolves to an encounter record carrying a patient identifier, which resolves in turn to the member attribute, enrollment, and utilization tables. All 2000 test messages linked to a patient record, yielding 1679 unique patients. Age and sex were complete, and race/ethnicity was recorded for 1674 (99.7%) of patients. Patient characteristics are reported in Table 2 and compared with national Medicaid benchmarks in Table 3. Equity-stratified sensitivity analyses by sex and by race/ethnicity were conducted as complete-case analyses restricted to messages with a non-missing value for the stratifying variable, and are framed as descriptive subgroup performance characterizations rather than as confirmatory equity claims.

**Table 2:** Characteristics of the patients who submitted the held-out real-world test messages

Characteristic	Patients (*N* = 1679)
Unique patients / test messages	1679 / 2000
Age (years): n known age	1679 (100.0%)
Mean (SD)	40.5 (16.5)
Median (IQR)	40.3 (29.6–53.9)
Age band: 0–17	136 (8.1%)
18–34	474 (28.2%)
35–49	548 (32.6%)
50–64	465 (27.7%)
65+	56 (3.3%)
Sex: n known sex	1679 (100.0%)
Female	1109 (66.1%)
Male	570 (33.9%)
Race/ethnicity: n known	1674 (99.7%)
African American	702 (41.9%)
Caucasian	697 (41.6%)
Unknown	138 (8.2%)
Hispanic	62 (3.7%)
Asian	30 (1.8%)
American Indian or Alaska Native	19 (1.1%)
Native Hawaiian or Other Pacific Islander	17 (1.0%)
Other	9 (0.5%)
State: Virginia	754 (45.0%)
Washington	432 (25.8%)
Ohio	488 (29.2%)
Prior utilization (annualized: events per enrolled-year): n with utilization linkage	1674 (99.7%)
Median observation window (months)	22
Emergency department visits — mean (median)	3.99 (1.71)
Emergency department visits — % with ≥ 1 during window	76.9%
Acute inpatient admissions — mean (median)	0.51 (0.00)
Acute inpatient admissions — % with ≥ 1 during window	26.3%
Outpatient/clinic visits — mean (median)	3.89 (1.02)
Outpatient/clinic visits — % with ≥ 1 during window	62.2%
Program engagement: Ever targeted for outreach	1670 (99.8%)
Ever activated (engaged with care team)	707 (42.2%)

Caption: Characteristics of the 1679 unique Medicaid patients who submitted the 2000 held-out test messages. Age is at 1 January 2026. Utilization is annualized as events per enrolled-year using enrolled days, because members contribute a median of 22 months of observation. All values are aggregate counts and percentages

**Table 3:** Study sample compared with national Medicaid benchmarks

Characteristic	This study	National Medicaid
Age: children 0–18 (% of enrollees < 65)	143 (8.5%)	47.8%
Age: adults 19–64 (% of enrollees < 65)	1480 (88.1%)	52.2%
Sex: female	1109 (66.1%)	56.9% (adults 19–64)
Race/ethnicity: Black / African American	702 (41.9%)	18.1%
Race/ethnicity: White / Caucasian	697 (41.6%)	40.0%
Race/ethnicity: Hispanic	62 (3.7%)	29.2%
Race/ethnicity: Asian	30 (1.8%)	5.2%
Race/ethnicity: American Indian or Alaska Native	19 (1.1%)	0.9%
Emergency department visits per enrolled-year	3.99	≈ 0.95 (adults)

Caption: The study sample set against published national Medicaid figures. Age, sex, and race/ethnicity benchmarks are Kaiser Family Foundation estimates based on the 2024 American Community Survey 1-Year Estimates [15]; the age denominator is enrollees under 65 years and the sex denominator is adults aged 19–64 years. The emergency department benchmark is for adults with Medicaid [16]. The sample is a care-management-engaged subpopulation and is not representative of national Medicaid; the differences shown are the basis for the generalizability limitation

Sensitivity to the residual missing-subgroup assumption was characterized for each architecture by Manski-style partial identification bounds on overall sensitivity [17, 18]. The bounds were constructed by computing the architecture’s observed sensitivity in the subgroup with a recorded value and then bracketing the overall sensitivity that would be observed if every hazard in the unrecorded subgroup were correctly detected (optimistic bound) or if no hazards in that subgroup were detected (conservative bound). For the high-sensitivity classifier underlying Policy A (logistic regression with TF-IDF features), the bounds are 0.758 (conservative) to 0.976 (optimistic), and the observed overall sensitivity (0.970) sits at the upper edge of the bound interval. Bounds for the remaining evaluated architectures are reported in Additional file 1.

### Sample size justification

The 2000-message held-out test set was sized to detect a between-architecture absolute sensitivity difference of 0.05 with 80% power at α = 0.05 (two-sided), under the planned-prevalence assumption of 8% hazard prevalence (observed prevalence 8.2%) and the planned-comparator-sensitivity assumption of 0.80. Under these parameters, the minimum hazard-positive subgroup required for the primary McNemar paired comparison was approximately 150 hazards; the realized hazard-positive subgroup was 165.

### Linguistic features

Three linguistic features were computed for every message. Flesch-Kincaid reading-level grade was computed using the textstat Python library (v0.7.0) on the raw message text after light pre-processing to strip non-printable characters and normalize whitespace. Colloquialism rate was computed as the proportion of words appearing in a 847-term colloquial-English lexicon seeded from published informal-language corpora and extended with Medicaid messaging-specific colloquialisms identified during the development-set labeling pass; the full lexicon and seed sources are released with the code repository. Abbreviation rate was computed by a rule-based detector requiring tokens to be ≤ 4 characters, non-stopword, non-single-letter pronoun, and either appearing ≥ 20 times in the corpus or matching a 312-term curated list of informal health abbreviations. Implicit-contextual-reference rate was computed as the proportion of clauses whose antecedent or referent is implicit rather than stated, identified by a rule-based annotator using spaCy (v3.5) coreference and dependency parsing followed by manual validation of the rule outputs on a 200-message random sample. Manual validation on the 200-message random sample showed Cohen kappa 0.73 for colloquialism detection, 0.86 for abbreviation detection, and 0.64 for implicit context identification. Full implementation details are reported in Additional file 1.

### Outcomes

The primary endpoint was sensitivity for hazard detection at each architecture’s calibrated operating threshold, with the 95% confidence interval computed by the Wilson score method [19].

Secondary endpoints: specificity (Wilson 95% confidence interval), positive predictive value, negative predictive value, F1 (bootstrap 95% confidence interval, 10,000 iterations, seed = 42), Matthews correlation coefficient (bootstrap), area under the receiver operating characteristic curve (Hanley-McNeil 95% confidence interval where calibrated probabilities were available), false negatives per 1,000 messages, action-recommendation appropriateness (3-category: appropriate, under-triage, over-triage on the 8-point action scale), equity-stratified sensitivity by sex and self-reported race/ethnicity.

Two operating-point targets were specified a priori. The first is the autonomous (physician-unassisted) triage benchmark, defined as sensitivity ≥ 0.80 AND specificity ≥ 0.80 simultaneously. The benchmark is anchored to the IDx-DR pivotal trial of an autonomous diabetic-retinopathy diagnostic system that received de novo authorization from the United States Food and Drug Administration as the first autonomous artificial intelligence diagnostic device cleared in any field of medicine, with pre-specified superiority endpoints of sensitivity > 85% and specificity > 82.5% and achieved values of sensitivity 87.2% (95% CI 81.8–91.2%) and specificity 90.7% (95% CI 88.3–92.7%) in the pivotal trial [20]. This target characterizes performance under hypothetical fully-autonomous deployment without clinician review. The second is a sensitivity-floor target with clinician review of flagged messages, defined as a sensitivity floor (≥ 0.80, ≥ 0.85, ≥ 0.90, and ≥ 0.95) at any specificity attainable on the operating curve, with the false-alarm volume routed to a clinician confirmatory review queue rather than blocking the workflow; this target instantiates the classical clinical-screening pattern (high-sensitivity screen followed by high-specificity confirmation) in which the cost of a missed hazard substantially exceeds the cost of a clinician review event. Both targets are reported.

### Architectures evaluated

Thirteen architecture configurations were evaluated on the held-out test set (Table 4). Nine were pre-specified primary configurations: rule-based guardrails, logistic regression with term-frequency features, XGBoost with sentence-embedding features, a multi-category classifier (the constellation architecture), two variants of a Conservative Q-Learning controller (sensitivity-optimized and reward-optimized), an action recommender (ActionHead), and two frontier large language models under a safety-augmented prompt (Claude Opus 4.7 and Gemini 3.1 Pro Preview). Four configurations were pre-specified in the protocol as optional robustness checks and are reported alongside the primary set: GPT-5.5 under the same safety-augmented prompt, and Claude Opus 4.7, GPT-5.5, and Gemini 3.1 Pro Preview each with retrieval-augmented generation. All configurations were trained and calibrated on the 570-message development subset and evaluated on the 2000-message held-out test set; no test-set message was used for hyperparameter selection, threshold calibration, or model selection. Brief descriptions follow; full technical specifications for each architecture are given in Additional file 1, Section C.

1. Rule-based guardrails: hand-crafted keyword and pattern rules [21].
2. Logistic regression with term frequency-inverse document frequency (TF-IDF) features [22].
3. XGBoost [23] over 768-dimension sentence-embedding features.
4. Constellation architecture: a multi-category classifier over hazard categories, reduced to a single binary hazard probability.
5. Conservative Q-Learning controller (sensitivity-optimized): a reinforcement-learning controller [24] over hazard-category probabilities whose reward penalizes missed hazards far more heavily than false alarms, operated at a threshold tuned for high sensitivity (Additional file 1, Section C).
6. Conservative Q-Learning controller (reward-optimized): the same controller with actions taken at the reward-maximizing choice.
7. ActionHead: a classifier that recommends one of eight ordinal triage actions.
8. Claude Opus 4.7 (safety-augmented): a frontier large language model prompted to prioritize patient safety and to err toward escalation; temperature 0.
9. Gemini 3.1 Pro Preview (safety-augmented): a frontier large language model under the same safety-augmented prompt; temperature 0.

**Table 4:** Per-architecture hazard detection metrics on the real-world test set

Architecture	TP / FN / TN / FP	Sensitivity (95% CI)	Specificity (95% CI)	PPV (95% CI)	NPV (95% CI)	F1 (95% CI)	MCC (95% CI)	AUROC (95% CI)	FN per 1,000 (95% CI)
ActionHead (action recommender)	70 / 95 / 1472 / 363	0.424 (0.351–0.501)	0.802 (0.783–0.820)	0.162 (0.130–0.199)	0.939 (0.926–0.950)	0.234 (0.189–0.279)	0.151 (0.099–0.204)	N/A*	47.5 (41.2–53.5)
Claude Opus 4.7 (safety-augmented)	49 / 116 / 1598 / 237	0.297 (0.233–0.371)	0.871 (0.855–0.885)	0.171 (0.132–0.219)	0.932 (0.919–0.943)	0.217 (0.167–0.268)	0.132 (0.077–0.189)	N/A*	58.0 (51.9–63.3)
Constellation architecture	11 / 154 / 1784 / 51	0.067 (0.038–0.115)	0.972 (0.964–0.979)	0.177 (0.102–0.290)	0.921 (0.908–0.932)	0.097 (0.046–0.152)	0.062 (0.004–0.125)	0.593 (0.546–0.640)	77.0 (73.0–79.4)
CQL controller (reward-optimized)	154 / 11 / 161 / 1674	0.933 (0.885–0.962)	0.088 (0.076–0.102)	0.084 (0.072–0.098)	0.936 (0.889–0.964)	0.155 (0.133–0.176)	0.021 (-0.021–0.057)	0.561 (0.514–0.608)	5.5 (3.1–9.5)
CQL controller (sensitivity-optimized)	4 / 161 / 1829 / 6	0.024 (0.009–0.061)	0.997 (0.993–0.999)	0.400 (0.168–0.687)	0.919 (0.906–0.930)	0.046 (0.011–0.092)	0.082 (0.004–0.162)	0.561 (0.514–0.608)	80.5 (77.5–81.7)
Gemini 3.1 Pro (safety-augmented)	3 / 162 / 1825 / 10	0.018 (0.006–0.052)	0.995 (0.990–0.997)	0.231 (0.082–0.503)	0.918 (0.906–0.930)	0.034 (0.000–0.075)	0.044 (-0.020–0.116)	N/A*	81.0 (78.2–82.0)
Rule-based guardrails	10 / 155 / 1803 / 32	0.061 (0.033–0.108)	0.983 (0.975–0.988)	0.238 (0.135–0.385)	0.921 (0.908–0.932)	0.097 (0.043–0.153)	0.083 (0.017–0.153)	0.503 (0.479–0.528)	77.5 (73.6–79.8)
Logistic regression + TF-IDF	160 / 5 / 170 / 1665	0.970 (0.931–0.987)	0.093 (0.080–0.107)	0.088 (0.076–0.102)	0.971 (0.935–0.988)	0.161 (0.139–0.183)	0.061 (0.030–0.086)	0.589 (0.546–0.633)	2.5 (1.1–5.7)
XGBoost + sentence-BERT	70 / 95 / 1328 / 507	0.424 (0.351–0.501)	0.724 (0.703–0.744)	0.121 (0.097–0.150)	0.933 (0.919–0.945)	0.189 (0.151–0.227)	0.090 (0.042–0.138)	0.606 (0.562–0.650)	47.5 (41.2–53.5)
Claude Opus 4.7 (RAG)	36 / 129 / 1684 / 151	0.218 (0.162–0.287)	0.918 (0.904–0.929)	0.193 (0.142–0.255)	0.929 (0.916–0.940)	0.205 (0.148–0.261)	0.128 (0.069–0.189)	N/A*	64.5 (58.8–69.1)
Gemini 3.1 Pro (RAG)	4 / 161 / 1798 / 37	0.024 (0.009–0.061)	0.980 (0.972–0.985)	0.098 (0.039–0.225)	0.918 (0.905–0.929)	0.039 (0.009–0.078)	0.008 (-0.034–0.059)	N/A*	80.5 (77.5–81.7)
GPT-5.5 (safety-augmented)	53 / 112 / 1583 / 252	0.321 (0.255–0.396)	0.863 (0.846–0.878)	0.174 (0.135–0.220)	0.934 (0.921–0.945)	0.226 (0.175–0.277)	0.141 (0.085–0.197)	N/A*	56.0 (49.8–61.5)
GPT-5.5 (RAG)	43 / 122 / 1630 / 205	0.261 (0.200–0.332)	0.888 (0.873–0.902)	0.173 (0.131–0.225)	0.930 (0.917–0.941)	0.208 (0.156–0.262)	0.124 (0.068–0.183)	N/A*	61.0 (55.1–66.0)

N/A*: AUROC is undefined for this architecture because the configured inference API returns a discrete hazard flag in a structured JSON output rather than a continuous probability scalar, leaving no score to sweep across thresholds. This affects the three frontier large language model configurations (Claude Opus 4.7, GPT-5.5, Gemini 3.1 Pro Preview) under both the safety-augmented and retrieval-augmented prompts, and the ActionHead action recommender, which emits a discrete action class rather than a calibrated hazard probability

Caption: Sensitivity, specificity, positive predictive value, negative predictive value, F1, Matthews correlation coefficient, area under the receiver operating characteristic curve (where calibrated probability output is available), and false negatives per 1,000 messages at each architecture’s calibrated operating threshold on the 2000-message held-out real-world test set. 95% confidence intervals: Wilson score method [12] for sensitivity, specificity, positive predictive value, and negative predictive value; parametric bootstrap (10,000 iterations, seed 42) for F1 and Matthews correlation coefficient; Hanley-McNeil for area under the receiver operating characteristic curve

### Single-turn screening evaluation

All architectures were evaluated in single-turn mode: each architecture received one patient message as input and produced one hazard prediction and one action recommendation. This corresponds to the “screening at message arrival” clinical use case, the decision the system makes upon receipt of an incoming message before any clarifying conversation occurs. Multi-turn dialogue extensions are out of scope for this analysis (see Limitations) and identified as future work.

### Comparability across architectures

The deployed decision support architecture (Conservative Q-Learning controller, constellation classifier, action recommender) and the classical supervised baselines (XGBoost with sentence-BERT, logistic regression with TF-IDF, rule-based guardrails) were fit on the 1,280-message development set with physician-adjudicated labels. The frontier large language models (Claude Opus 4.7, GPT-5.5, Gemini 3.1 Pro Preview) were evaluated zero-shot under a safety-augmented system prompt without parameter updates. The retrieval-augmented variants of each frontier model additionally received eight stratified labeled neighbors from the same development corpus in-context but did not update model weights. Head-to-head differences between the supervised pipeline and the frontier-LLM configurations therefore confound algorithm class with training-data exposure. The retrieval-augmented frontier-LLM configurations are the closest matched comparators, providing both in-context labeled examples and identical zero-shot model weights to the bare safety-augmented variants. The relative within-pipeline rankings (deployed Conservative Q-Learning controller vs. supervised baselines, and safety-augmented vs. retrieval-augmented within each frontier-LLM vendor) are interpretable independent of this confound.

### Sensitivity analyses

Three additional analyses were specified a priori as sensitivity extensions of the primary head-to-head comparison.

First, multi-model consensus rules were computed from the existing per-message frontier-model predictions: Claude-only, Gemini-only, OR-rule of either flagging, and AND-rule of both flagging.

Second, retrieval-augmented generation was added to the safety-augmented configuration of each of the three frontier large language models (Claude Opus 4.7, GPT-5.5, and Gemini 3.1 Pro Preview). At inference, the top-eight nearest-neighbor labeled examples from the 1,280-example development corpus, retrieved by sentence-BERT cosine similarity and stratified by class, were supplied to each model as in-context evidence.

Third, a four-component guardrail composition was operationalized as a synthesis of three current health AI assurance frameworks: the assurance-laboratory evaluation framework of Shah and colleagues [25], the principles articulated in the Coalition for Health AI Blueprint for Trustworthy AI Implementation Guidance and Assurance for Healthcare [26], and the organizational governance approach of the Health AI Partnership community of practice [27]. The four components were: (1) a clinical-safety system prompt at the language-model layer; (2) a content-moderation filter pre-screen at the message-ingestion layer; (3) retrieval-augmented generation grounding the language model in labeled population-relevant exemplars; and (4) a learned hazard-classifier pre-screen filtering candidate messages before language-model evaluation. Components 1 and 3 were realized as the safety-augmented Claude Opus 4.7 with retrieval-augmented generation. Component 2 was modeled as a pass-through on the clinically-themed test-set messages, since in deployment the component would gate at message ingestion and would not exclude any messages in the present test set. Component 4 was realized as one of seven evaluated supervised classifiers, with the gate and language model composed under an AND-rule cascade requiring concordance between the two stages.

Two operating-point analyses for use with clinician review of flagged messages were also specified a priori. The first was a sensitivity-floor analysis: across the full pool of evaluated configurations (single tools at every observed threshold, ensembles, cascades, multi-model consensus rules, and retrieval-augmented generation), the configuration achieving the maximum specificity at each of four sensitivity floors (0.80, 0.85, 0.90, 0.95) was identified. The second was a disagreement-stratified workflow: the count of tools flagging each message was treated as an uncertainty signal, with messages flagged by zero or one of the thirteen architectures considered candidates for autonomous-benign disposition and messages flagged by two or more routed to clinician review.

### Statistical analysis

All paired comparisons used McNemar’s test [28] restricted to hazard-positive cases, with Hochberg step-up correction [29] for family-wise error rate control across the 78 pairwise comparisons.

Cross-set sensitivity comparisons (physician-scripted versus real-world test sets) used a parametric bootstrap with 10,000 iterations and seed 42. In each iteration, sensitivities for both sets were independently resampled from binomial distributions parameterized by the observed proportions and sample sizes.

Stratified equity analyses used the same Wilson confidence intervals within each subgroup, with the equalized odds difference (maximum minus minimum subgroup sensitivity) reported.

#### Deviations from the pre-registered protocol

The protocol specified six primary configurations and fifteen primary pairwise comparisons. The evaluated set was expanded to the thirteen configurations reported here (Table 4), and paired comparisons were computed across all 78 architecture pairs with Hochberg control. GPT-5.5 and the retrieval-augmented variants were the optional robustness checks anticipated in the protocol. No change was made to the primary endpoint, the operating-point targets, the calibration-freezing rule, or the analysis seed.

### Reporting framework and reproducibility

The study follows TRIPOD + AI [30] and DECIDE-AI [31] reporting guidelines. The complete TRIPOD + AI checklist and the complete DECIDE-AI checklist both appear in Additional file 1, Section A.

Per-message predictions for every architecture × every test record are retained by the corresponding author and available on request for verification. Patient message text remains under HIPAA data-use agreement and is available to bona-fide researchers on request to the corresponding author.

The code repository at https://github.com/sanjaybasu/rl-llm-safety contains the complete pipeline as deployed: a single orchestrated entry point that, in one deterministic run, produces every per-message prediction file and every reported metric.

### De-identification and adjudicator role

Patient messages were de-identified by HIPAA Safe Harbor methodology prior to analysis. No identifiable patient data or direct verbatim quotations appear in any manuscript figure, table, or supplementary file. Reviewing physicians performed adjudication as part of their salaried clinical-network duties and received no additional study-specific compensation. The IRB approval and consent waiver for this study are reported in the Declarations section.

## Results

### Population characteristics

The 2000 held-out test messages were submitted by 1679 unique Medicaid patients during January 2023 through November 2025. Hazard prevalence in the test set was 8.2% (165 hazards across 2000 messages), with the remaining 1835 messages adjudicated as benign by all three reviewing physicians. Patients had a mean age of 40.5 (16.5) years; 1109 (66.1%) were female; and they were distributed across Virginia (754 (45.0%)), Washington (432 (25.8%)), and Ohio (488 (29.2%)). Prior utilization was high: a mean of 3.99 emergency department visits per enrolled-year, with 76.9% of patients having at least one emergency department visit during the observation window. Full patient characteristics are reported in Table 2, and Table 3 sets them against national Medicaid benchmarks. The linguistic profile of the test set departed substantially from the profile of physician-scripted comparison scenarios on two of four measured dimensions and in the opposite direction on the other two (Table 1). These linguistic features describe the message text itself, that is, the input each tool received, rather than any attribute of the patient. Real-world messages were written at a lower reading level than physician-scripted scenarios (mean Flesch-Kincaid grade 4.6 versus 5.8) and contained colloquialisms more often (59.1% versus 19.5%); abbreviations (24.5% versus 36.6%) and implicit contextual references (7.8% versus 29.3%) were less frequent, reflecting the deliberate medical shorthand of scripted scenarios. The hazard categories (behavioral-health symptoms, substance-use escalation, obstetric concerns, pediatric overdose, metabolic emergencies, and others) reflect the natural case mix of a Medicaid managed care messaging service rather than the curated distribution of a standardized benchmark.

### Hazard detection performance

Table 4 reports per-architecture sensitivity, specificity, positive predictive value, negative predictive value, F1, Matthews correlation coefficient, area under the receiver operating characteristic curve, and false negatives per 1,000 messages on the 2000-message held-out test set.

### Operating-point analysis

Table 5 reports sensitivity at matched specificity targets for each architecture for which calibrated probability output is available. Both the target specificity (the operating point at which each architecture is requested to operate) and the achieved specificity (the actual specificity observed at the threshold required to attain the target) are displayed alongside the sensitivity attained at that operating point. By construction, the rows in Table 5 are derived from the same per-message prediction files as Table 4, with no architecture’s operating curve drawn from a different prediction run. Receiver operating characteristic monotonicity (sensitivity non-decreasing as specificity decreases) is verified for every architecture with calibrated probability output as a pre-submission pipeline assertion.

**Table 5:** Operating-point analysis: sensitivity at matched specificity targets for architectures with calibrated probability output

Architecture	Sens at Spec ≥ 0.70	Sens at Spec ≥ 0.73	Sens at Spec ≥ 0.80	Sens at Spec ≥ 0.90	Sens at Spec ≥ 0.95
Constellation architecture	0.430 (0.357–0.507)	0.406 (0.334–0.482)	0.327 (0.260–0.402)	0.218 (0.162–0.287)	0.127 (0.085–0.187)
CQL controller (reward-optimized)	0.412 (0.340–0.488)	0.370 (0.300–0.446)	0.291 (0.227–0.364)	0.139 (0.095–0.200)	0.109 (0.070–0.166)
CQL controller (sensitivity-optimized)	0.412 (0.340–0.488)	0.370 (0.300–0.446)	0.291 (0.227–0.364)	0.139 (0.095–0.200)	0.109 (0.070–0.166)
Rule-based guardrails	0.061† (0.033–0.108)	0.061† (0.033–0.108)	0.061† (0.033–0.108)	0.061† (0.033–0.108)	0.061† (0.033–0.108)
Logistic regression + TF-IDF	0.424 (0.351–0.501)	0.394 (0.323–0.470)	0.303 (0.238–0.377)	0.139 (0.095–0.200)	0.061 (0.033–0.108)
XGBoost + sentence-BERT	0.461 (0.386–0.537)	0.418 (0.346–0.494)	0.327 (0.260–0.402)	0.133 (0.090–0.194)	0.091 (0.056–0.145)

Caption: For each architecture with calibrated probability output, the threshold that achieves the target specificity is identified on the architecture’s receiver operating characteristic curve, and the sensitivity attained at that threshold is reported with the achieved specificity displayed alongside the target. The receiver operating characteristic curves derive from the same per-message prediction files as Table 4; monotonicity (sensitivity non-decreasing as specificity decreases) is enforced as a pipeline assertion

### Paired comparisons

**Fig. 1:**
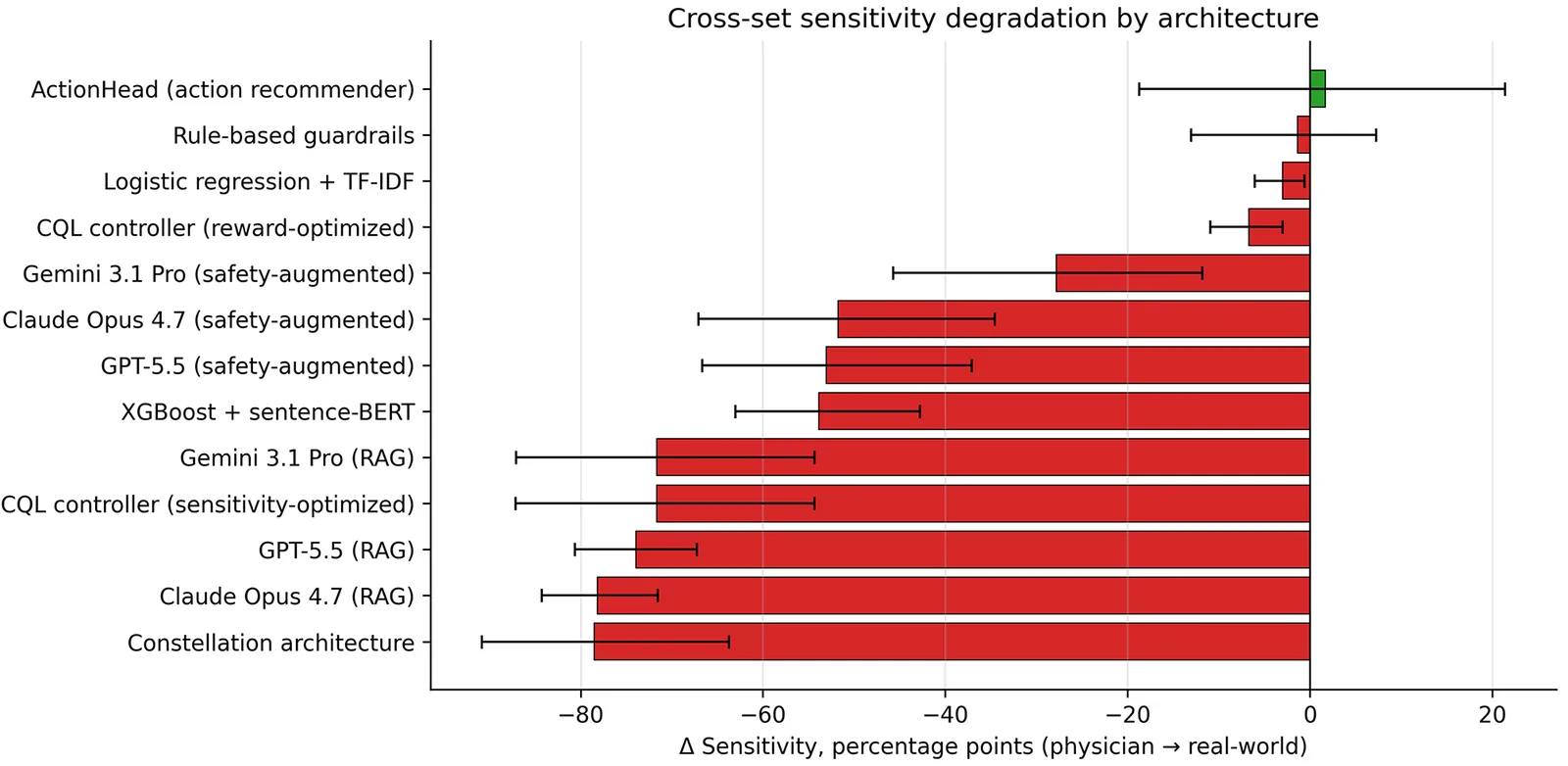
Per-architecture sensitivity change from physician-scripted to real-world Medicaid test sets. Caption: Each architecture’s sensitivity on the physician-scripted 41-case comparison set is plotted against its sensitivity on the held-out 2000-message real-world test set. The diagonal indicates equal performance across populations; architectures below the diagonal lose sensitivity when moving from the physician comparison scenarios to the real-world Medicaid messaging population. Error bars reflect Wilson 95% confidence intervals

Figure 1 visualizes paired sensitivity differences between the deployed clinical decision support architecture variants and the frontier large language model comparators on the held-out test set. Each pairwise comparison was tested with McNemar’s test [28] restricted to hazard-positive cases, with Hochberg step-up correction [29] applied across the 78 pairwise comparisons. The full pairwise discordant-pair matrix is reported in Additional file 1, Table S5.

### Action recommendation appropriateness

**Fig. 2:**
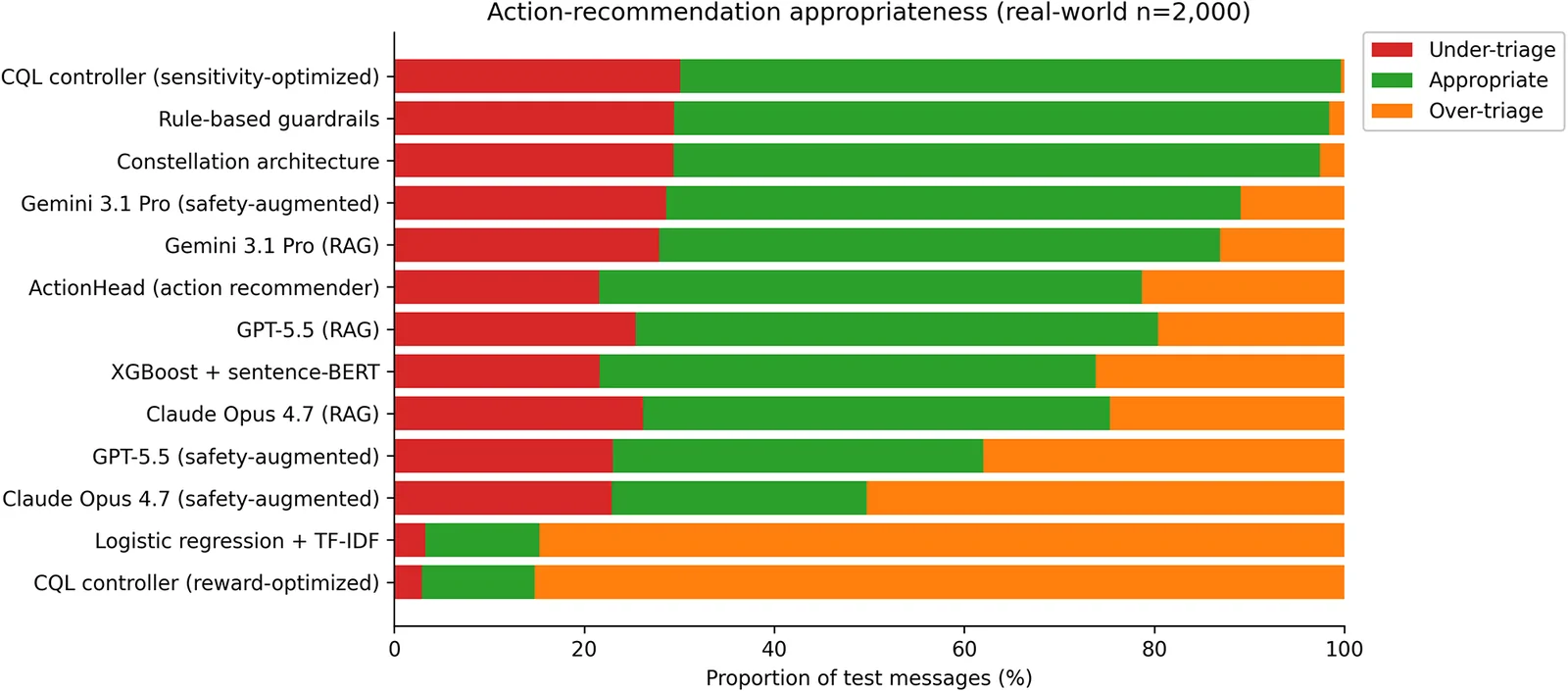
Action recommendation appropriateness on the real-world test set, by architecture.Caption: Stacked-bar chart of the proportion of recommendations classified as appropriate (matching the physician-adjudicated reference action), under-triage (recommending a less urgent action than the reference), or over-triage (recommending a more urgent action) on the 8-point ordinal action scale, on the real-world test set

Figure 2 displays the per-architecture proportion of recommendations classified as appropriate, under-triage (recommending a less urgent action than the physician-adjudicated reference), or over-triage (recommending a more urgent action than the reference), on the 8-point ordinal action scale. Additional file 1, Table S7 reports the underlying counts, the reference action distribution, and the agreement structure between each architecture and the reference.

### Equity-stratified analysis

Sensitivity stratified by sex and by self-reported race/ethnicity is reported with Wilson 95% confidence intervals [19] within each subgroup, with the equalized odds difference (maximum minus minimum subgroup sensitivity) reported per architecture. Subgroups with fewer than 30 hazard-positive cases are flagged as insufficiently powered for reliable subgroup inference and are reported with explicit small-sample warnings rather than suppressed.

### Threshold-optimized single-architecture analysis

To distinguish discriminative-capacity limitations from threshold-calibration artifacts, the operating curve of every architecture with calibrated probability output was swept and the F1-maximizing and Matthews-correlation-coefficient-maximizing operating points were extracted (Table 6). Across all six architectures with calibrated probabilities, the maximum F1 attained by any single architecture at any threshold was 0.20 (XGBoost with sentence-BERT, F1 0.201 at threshold-tuned sensitivity 0.412 and specificity 0.759), and the maximum Matthews correlation coefficient was 0.11 (constellation classifier, 0.109). At no operating point on any architecture’s receiver operating characteristic curve was sensitivity ≥ 0.80 AND specificity ≥ 0.80 simultaneously achieved (Table 6). The architecture with the highest balanced operating point was XGBoost with sentence-BERT at sensitivity 0.594 and specificity 0.592, the point closest to the autonomous (physician-unassisted) triage benchmark. This remains well below the autonomous-deployment benchmark used here (sensitivity 0.80 or higher and specificity 0.80 or higher), which is anchored to the IDx-DR autonomous-diagnostic-AI pivotal trial [20]. The Conservative Q-Learning controller variants are bounded by the same underlying receiver operating characteristic envelope: their separation in Table 4 reflects threshold calibration on the asymmetric reward design rather than fundamentally different discriminative capacity.

**Table 6:** Threshold-optimized single-architecture operating points and autonomous-benchmark reachability

Architecture	F1-max (95% CI)	Sens at F1-max (95% CI)	Spec at F1-max (95% CI)	MCC-max (95% CI)	Sens at MCC-max (95% CI)	Spec at MCC-max (95% CI)	Reached autonomous benchmark?	Sens/Spec at closest point (95% CI)
Constellation architecture	0.197 (0.161–0.234)	0.376 (0.305–0.452)	0.781 (0.761–0.799)	0.109 (0.057–0.160)	0.218 (0.162–0.287)	0.904 (0.890–0.917)	No	0.564 / 0.563 (sens CI 0.487–0.637; spec CI 0.540–0.585)
CQL controller (reward-optimized)	0.175 (0.146–0.206)	0.412 (0.340–0.488)	0.704 (0.682–0.724)	0.096 (0.026–0.166)	0.067 (0.038–0.115)	0.983 (0.976–0.988)	No	0.539 / 0.540 (sens CI 0.463–0.614; spec CI 0.517–0.563)
CQL controller (sensitivity-optimized)	0.175 (0.146–0.206)	0.412 (0.340–0.488)	0.704 (0.682–0.724)	0.096 (0.026–0.166)	0.067 (0.038–0.115)	0.983 (0.976–0.988)	No	0.539 / 0.540 (sens CI 0.463–0.614; spec CI 0.517–0.563)
Rule-based guardrails	0.153 (0.152–0.153)	1.000 (0.977–1.000)	0.002 (0.001–0.006)	0.083 (0.018–0.152)	0.061 (0.033–0.108)	0.983 (0.975–0.988)	No	0.061 / 0.983 (sens CI 0.033–0.108; spec CI 0.975–0.988)
Logistic regression + TF-IDF	0.183 (0.152–0.217)	0.412 (0.340–0.488)	0.723 (0.702–0.743)	0.095 (0.061–0.128)	0.873 (0.813–0.915)	0.281 (0.261–0.302)	No	0.551 / 0.564 (sens CI 0.475–0.625; spec CI 0.541–0.586)
XGBoost + sentence-BERT	0.201 (0.166–0.239)	0.412 (0.340–0.488)	0.759 (0.738–0.778)	0.108 (0.058–0.161)	0.412 (0.340–0.488)	0.759 (0.738–0.778)	No	0.594 / 0.592 (sens CI 0.518–0.666; spec CI 0.569–0.614)

Caption: For each architecture with calibrated probability output, the F1-maximizing and Matthews-correlation-coefficient-maximizing operating points on the real-world test set receiver operating characteristic curve are reported, along with the closest-to-symmetric-benchmark operating point and a binary indicator of whether the architecture’s receiver operating characteristic curve passes through the sensitivity ≥ 0.80 AND specificity ≥ 0.80 region. Threshold-optimized analysis is post hoc: the thresholds reported here are selected on the real-world test set operating curve and characterize the upper bound of threshold-tuning achievable performance; clinical deployment requires threshold selection on a held-out validation set or prospective calibration. The headline result is that no architecture reaches the autonomous (physician-unassisted) triage benchmark at any operating point on its receiver operating characteristic curve

### Ensemble architectures and cascade architectures

**Fig. 3:**
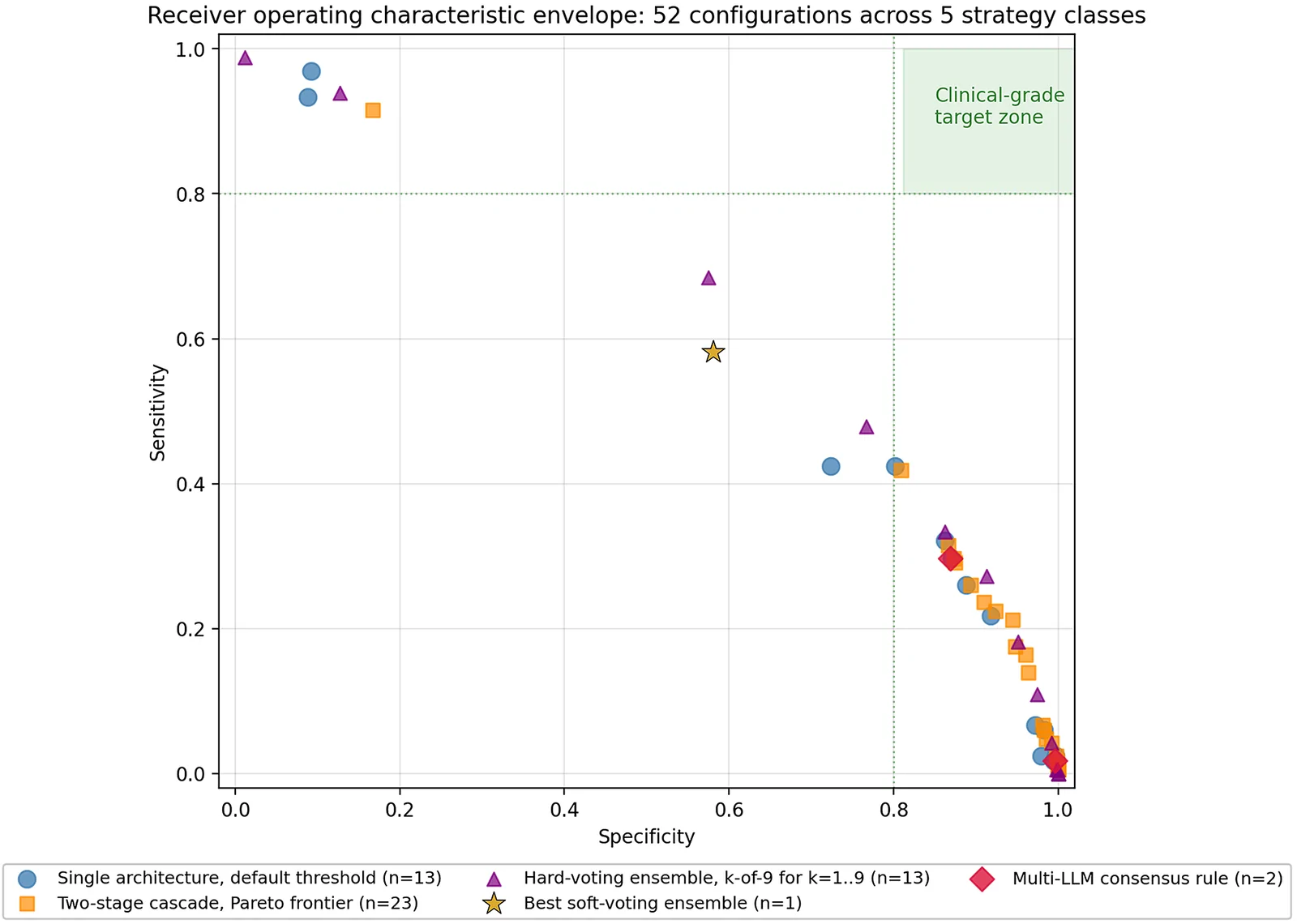
Receiver operating characteristic envelope across all evaluated strategies on the real-world test set. Caption: Each marker is one configuration. Markers of the same shape and color are different configurations within the same strategy class. The legend reports the number of configurations per class: 13 single-architecture default-threshold operating points (blue circles, one per architecture), 23 two-stage cascade Pareto-frontier pairs (orange squares), 13 hard-voting k-of-13 ensemble configurations for k from 1 to 13 (purple triangles), 1 best soft-voting ensemble (gold star), and 2 multi-model consensus rules (red diamonds). Many configurations cluster near the high-specificity / low-sensitivity corner, where overlapping markers of different shapes may look like a single composite marker. The shaded green zone in the upper-right is the autonomous (physician-unassisted) triage benchmark (sensitivity 0.80 or higher and specificity 0.80 or higher). No configuration reaches the benchmark zone, the structural finding of this study

Two architectural-combination strategies were evaluated against the autonomous (physician-unassisted) triage benchmark. First, 217 ensemble configurations were enumerated, combining all 13 architectures via hard-voting k-of-13 rules (k from 1 to 13), soft-voting at 101 thresholds with unweighted and F1-weighted means of the calibrated probability outputs, and top-three AND/OR rules. None achieved sensitivity 0.80 or higher and specificity 0.80 or higher simultaneously. The best balanced ensemble was the hard-voting three-of-13 rule (sensitivity 0.685, specificity 0.575, F1 0.214). The best F1-weighted soft-voting configuration reached sensitivity 0.582 and specificity 0.581 at the threshold-swept optimum. Second, all 156 ordered two-stage AND-rule cascade configurations were enumerated, with the Pareto frontier reported in Table 7 and the full matrix in Additional file 1, Table S3. The cascade pairing the Conservative Q-Learning controller (reward-optimized) with the safety-augmented Claude Opus 4.7 attained sensitivity 0.297 and specificity 0.874, improving specificity at the cost of sensitivity as expected for AND-rule cascades. The cascade pairing the action recommender with the logistic regression screen attained the highest cascade F1 (0.236) and highest Matthews correlation coefficient (0.154). No cascade configuration reached the autonomous (physician-unassisted) triage benchmark. The architectural-combination findings extend the single-architecture result: neither ensembling nor cascading the evaluated architectures closes the population gap. Figure 3 visualizes the joint distribution of sensitivity and specificity across all evaluated strategies (single architectures, ensembles, cascades, and multi-model consensus rules); no marker reaches the upper-right benchmark region defined by sensitivity ≥ 0.80 and specificity ≥ 0.80.

**Table 7:** Cascade architectures: Pareto frontier of two-stage AND-rule configurations on the real-world test set

Cascade (Stage 1 × Stage 2)	Sensitivity (95% CI)	Specificity (95% CI)	PPV (95% CI)	F1 (95% CI)	MCC (95% CI)	FN per 1,000 (95% CI)
CQL reward-opt × LogReg + TF-IDF	0.915 (0.863–0.949)	0.167 (0.151–0.185)	0.090 (0.077–0.105)	0.164 (0.156–0.171)	0.062 (0.026–0.094)	7.0 (4.2–11.3)
ActionHead × LogReg + TF-IDF	0.418 (0.346–0.494)	0.809 (0.791–0.827)	0.165 (0.132–0.203)	0.236 (0.194–0.278)	0.154 (0.099–0.209)	48.0 (41.7–54.0)
CQL reward-opt × GPT-5.5	0.315 (0.249–0.390)	0.867 (0.851–0.882)	0.176 (0.137–0.223)	0.226 (0.179–0.270)	0.141 (0.087–0.194)	56.5 (50.4–61.9)
Claude Opus 4.7 × CQL reward-opt	0.297 (0.233–0.371)	0.874 (0.858–0.888)	0.174 (0.134–0.223)	0.220 (0.174–0.266)	0.135 (0.081–0.188)	58.0 (51.9–63.3)
Claude Opus 4.7 × LogReg + TF-IDF	0.291 (0.227–0.364)	0.875 (0.859–0.889)	0.173 (0.133–0.221)	0.217 (0.172–0.263)	0.132 (0.078–0.185)	58.5 (52.4–63.8)
CQL reward-opt × GPT-5.5 + RAG	0.261 (0.200–0.333)	0.894 (0.879–0.907)	0.181 (0.137–0.235)	0.213 (0.165–0.260)	0.131 (0.077–0.184)	61.0 (55.1–66.0)
Claude Opus 4.7 × GPT-5.5	0.236 (0.178–0.307)	0.909 (0.895–0.922)	0.190 (0.142–0.249)	0.211 (0.160–0.260)	0.132 (0.078–0.188)	63.0 (57.2–67.8)
GPT-5.5 + RAG × GPT-5.5	0.224 (0.167–0.294)	0.924 (0.911–0.935)	0.210 (0.157–0.276)	0.217 (0.165–0.271)	0.144 (0.088–0.202)	64.0 (58.3–68.7)
ActionHead × GPT-5.5	0.212 (0.157–0.281)	0.944 (0.933–0.954)	0.256 (0.190–0.334)	0.232 (0.174–0.291)	0.171 (0.108–0.233)	65.0 (59.3–69.6)
Claude 4.7 + RAG × GPT-5.5	0.176 (0.125–0.241)	0.948 (0.936–0.957)	0.232 (0.167–0.313)	0.200 (0.140–0.263)	0.140 (0.076–0.208)	68.0 (62.6–72.2)
ActionHead × GPT-5.5 + RAG	0.164 (0.115–0.228)	0.960 (0.950–0.968)	0.270 (0.193–0.364)	0.204 (0.141–0.272)	0.156 (0.089–0.231)	69.0 (63.7–73.0)
ActionHead × Claude 4.7 + RAG	0.139 (0.095–0.201)	0.964 (0.954–0.971)	0.256 (0.177–0.354)	0.180 (0.120–0.247)	0.137 (0.072–0.212)	71.0 (66.0–74.7)
Constellation × XGBoost+SBERT	0.067 (0.038–0.115)	0.982 (0.974–0.987)	0.244 (0.142–0.387)	0.105 (0.049–0.164)	0.089 (0.020–0.161)	77.0 (73.0–79.4)
CQL reward-opt × Guardrails	0.061 (0.033–0.108)	0.983 (0.976–0.988)	0.244 (0.138–0.393)	0.097 (0.041–0.154)	0.085 (0.017–0.154)	77.5 (73.6–79.8)
Constellation × GPT-5.5	0.049 (0.025–0.093)	0.986 (0.979–0.990)	0.235 (0.124–0.400)	0.080 (0.031–0.137)	0.073 (0.007–0.142)	78.5 (74.9–80.5)
Guardrails × XGBoost+SBERT	0.042 (0.021–0.085)	0.992 (0.987–0.995)	0.318 (0.164–0.527)	0.075 (0.023–0.131)	0.090 (0.017–0.167)	79.0 (75.5–80.8)
CQL sens-opt × XGBoost+SBERT	0.024 (0.009–0.061)	0.998 (0.994–0.999)	0.500 (0.215–0.785)	0.046 (0.012–0.091)	0.096 (0.010–0.179)	80.5 (77.5–81.7)
Gemini 3.1 Pro × XGBoost+SBERT	0.018 (0.006–0.052)	0.999 (0.996–1.000)	0.600 (0.231–0.882)	0.035 (0.000–0.080)	0.094 (-0.009–0.170)	81.0 (78.2–82.0)
CQL sens-opt × GPT-5.5 + RAG	0.012 (0.003–0.043)	1.000 (0.997–1.000)	0.667 (0.208–0.939)	0.024 (0.000–0.059)	0.082 (-0.009–0.167)	81.5 (78.9–82.2)
Constellation × Guardrails	0.012 (0.003–0.043)	1.000 (0.997–1.000)	0.667 (0.208–0.939)	0.024 (0.000–0.059)	0.082 (-0.009–0.167)	81.5 (78.9–82.2)
Constellation × Gemini 3.1 Pro	0.012 (0.003–0.043)	1.000 (0.997–1.000)	0.667 (0.208–0.939)	0.024 (0.000–0.059)	0.082 (-0.009–0.167)	81.5 (78.9–82.2)
CQL sens-opt × Guardrails	0.006 (0.001–0.034)	1.000 (0.998–1.000)	1.000 (0.207–1.000)	0.012 (0.000–0.036)	0.075 (0.075–0.149)	82.0 (79.7–82.4)
CQL sens-opt × Gemini 3.1 Pro	0.006 (0.001–0.034)	1.000 (0.998–1.000)	1.000 (0.207–1.000)	0.012 (0.000–0.036)	0.075 (0.075–0.149)	82.0 (79.7–82.4)

Caption: Each row reports a Stage 1 architecture (high-recall screen) paired with a Stage 2 architecture (high-precision filter), with the final positive decision requiring concordance between both stages. Sensitivity, specificity, positive predictive value, F1, and Matthews correlation coefficient are computed on the AND-rule cascade output. Pareto-frontier pairs (those not dominated on both sensitivity and specificity by any other pair) are listed; no cascade configuration attained sensitivity ≥ 0.80 AND specificity ≥ 0.80 simultaneously. The cascade pairing the asymmetric-reward Conservative Q-Learning controller with the safety-augmented Claude Opus 4.7 frontier large language model is the highest-specificity pair in the cascade matrix that retains non-trivial sensitivity. The full 156-configuration cascade matrix is provided in Additional file 1, Table S3

### Sensitivity analyses: multi-model consensus, retrieval-augmented generation, and four-component guardrail composition

Two additional closing-the-gap interventions identified by a structured literature review of 2024–2026 clinical artificial intelligence safety evaluations were evaluated against the autonomous (physician-unassisted) triage benchmark. First, multi-model consensus rules (Claude Opus 4.7 only, Gemini 3.1 Pro Preview only, OR-rule of either flagging, AND-rule of both flagging) were computed from the existing per-message predictions. The OR-rule consensus attained sensitivity 0.297 and specificity 0.869, virtually identical to Claude alone because the Gemini-positive set was nearly a subset of the Claude-positive set on this population. The AND-rule consensus attained sensitivity 0.018 and specificity 0.996, dominated by Gemini’s low single-model sensitivity. No multi-model consensus rule reached the autonomous (physician-unassisted) triage benchmark.

Second, retrieval-augmented generation over the 1,280-example labeled training corpus was evaluated as a separate closing-the-gap intervention across all three frontier large language model providers (Claude Opus 4.7, GPT-5.5, and Gemini 3.1 Pro Preview). Each model received the top-eight stratified-by-class nearest-neighbor training examples as in-context evidence at inference time, retrieved by sentence-BERT cosine similarity. All three providers showed a consistent dissociation between the physician-scripted 41-case comparison set and the real-world Medicaid messaging test set. On the physician-scripted set, retrieval-augmented generation raised sensitivity above the safety-augmented baseline for every vendor (Claude Opus 4.7: 0.815 to 1.000; GPT-5.5: 0.852 to 1.000; Gemini 3.1 Pro: 0.296 to 0.741), with the corresponding F1 lifts (Claude 0.800 to 0.900; GPT-5.5 0.821 to 0.947; Gemini 0.432 to 0.851) confirming the published RAG benefit on prototypically-presented clinical scenarios [12]. On the real-world Medicaid messaging set, the same retrieval-augmented configurations attained sensitivity 0.218 (Claude, down from 0.297 without retrieval), 0.261 (GPT-5.5, down from 0.321), and 0.024 (Gemini, essentially unchanged from 0.018). Retrieval-augmented generation closed the gap on prototypically-presented physician scenarios for all three vendors but failed to close the gap on the Medicaid messaging population for any vendor. One interpretation is that retrieval over the 1,280-example labeled training corpus returns informative neighbors when the test message resembles training-set patterns (the physician-scripted scenarios were constructed to be representative of canonical hazard presentations), but returns lower-information or distribution-mismatched neighbors when the test message exhibits the colloquialism-rich, abbreviation-laden, implicit-context patterns characteristic of the real-world Medicaid messaging population. The cross-vendor replication of this dissociation strengthens the interpretation by ruling out a single-vendor model-specific artifact. The result is consistent with the literature-review finding that no published 2024–2026 study has demonstrated sensitivity ≥ 0.80 AND specificity ≥ 0.80 for free-text hazard detection on a Medicaid or low-literacy population.

Table S8 synthesizes the best operating points achievable under each strategy and reports whether any strategy reached the autonomous (physician-unassisted) triage benchmark on the real-world test set.

Third, the four-component guardrail composition specified in Methods (a clinical-safety prompt, a content-moderation pre-screen, retrieval-augmented generation, and a learned hazard-classifier gate, synthesizing the assurance frameworks of Shah and colleagues [25], the Coalition for Health AI [26], and the Health AI Partnership [27]) was evaluated. Across all seven learned-classifier gates, no configuration reached sensitivity 0.80 or higher with specificity 0.80 or higher on the real-world test set; the operating point was dominated by the second-stage language model’s sensitivity floor, and the gate further reduced sensitivity by requiring cross-stage concordance (Additional file 1, Table S9). The multi-component guardrail compositions described in the assurance and governance literature therefore appear to require population-specific extension.

### Operating-point analysis under a sensitivity-floor target

The autonomous (physician-unassisted) triage benchmark (sensitivity 0.80 or higher and specificity 0.80 or higher simultaneously) was not reached by any evaluated configuration on the real-world Medicaid messaging population. The complementary question, what is achievable at a sensitivity-floor target appropriate for use with clinician review of flagged messages, was characterized by two analyses (Table 8). First, a sensitivity-floor analysis swept the full pool of evaluated configurations (single architectures with calibrated probabilities at all observed thresholds, ensembles, cascades, multi-model consensus rules, and retrieval-augmented generation) and identified the configuration achieving the maximum specificity at each of four sensitivity floors (≥ 0.80, ≥ 0.85, ≥ 0.90, ≥ 0.95). The configuration meeting sensitivity 0.85 or higher with the highest achievable specificity was a threshold-tuned logistic regression with TF-IDF features, attaining sensitivity 0.855 and specificity 0.282. Operationally, this is a high-recall first-stage screen that forwards approximately 72% of test-set messages to second-stage clinician review. Second, a disagreement-stratified clinician-review workflow was characterized by treating the count of architectures flagging each message as an uncertainty signal: messages flagged by zero or one of the thirteen evaluated architectures (244 of 2,000 messages; 12.2% of the test set) had a hazard prevalence of 4.1% in this stratum and are candidates for autonomous-benign disposition; messages flagged by two or more architectures (1,756 of 2,000; 87.8% of the test set) require clinician review and collectively capture 155 of 165 hazards (sensitivity 0.939). The empirical alert volume and residual miss rate per 1,000 messages for both policies, alongside the best balanced single-architecture, ensemble, and cascade reference points, are reported in Table 9. The disagreement-stratified workflow attains higher headline sensitivity than any single autonomous configuration but at the cost of a clinician review load that scales with 88% of the messaging volume. Both configurations meeting the pre-specified sensitivity-floor target are reported as operating points, the operationally-relevant operating pattern for safety-critical messaging triage with asymmetric harm; the research agenda below addresses the residual gap to the autonomous (physician-unassisted) triage benchmark.

**Table 8:** Deployment-grade configurations: sensitivity-floor analysis and disagreement-stratified clinician-review workflow

**Panel A — Sensitivity-floor target: max specificity achievable subject to sensitivity threshold**				
**Sensitivity floor**	***N*** **configurations meeting floor**	**Max specificity at floor (95% CI)**	**Winning configuration**	**Sens (winning) (95% CI)**
≥ 0.80	2911	0.359 (0.337–0.381)	single_architecture_threshold_sweep / LogReg + TF-IDF@thresh	0.800 (0.732–0.854)
≥ 0.85	2242	0.282 (0.262–0.303)	single_architecture_threshold_sweep / LogReg + TF-IDF@thresh	0.855 (0.793–0.900)
≥ 0.90	1563	0.191 (0.173–0.209)	single_architecture_threshold_sweep / XGBoost+SBERT@thresh	0.903 (0.848–0.939)
≥ 0.95	887	0.098 (0.085–0.112)	single_architecture_threshold_sweep / XGBoost+SBERT@thresh	0.952 (0.907–0.975)
**Panel B ** **—** ** Disagreement-stratified clinician-review policy**				
**Stratum (n architectures flagging)**	**Recommended policy**	**N messages**	**N hazards (true)**	**Share of total messages**
0–1 flagging (autonomous benign)	no action	244	10	12.2%
2–3 flagging (clinician review)	clinician review	1250	76	62.5%
4–6 flagging (clinician review)	clinician review	386	49	19.3%
7–13 flagging (autonomous escalation)	escalate	120	30	6.0%

Caption: Panel A — for each of four sensitivity floors (≥ 0.80, ≥ 0.85, ≥ 0.90, ≥ 0.95), the configuration achieving the maximum specificity at or above that floor across the full pool of evaluated configurations (single architectures at all observed thresholds, ensembles, cascades, multi-model consensus rules, retrieval-augmented generation). Panel B — distribution of the 2000 real-world test-set messages across agreement strata (number of architectures flagging each message), with the policy recommendation for clinician review or autonomous disposition at each stratum, the resulting hazard prevalence within the stratum, and the share of total message volume comprising the stratum

**Table 9:** Two deployment policies vs. individual-component baselines: empirical alert volume and residual miss rate per 1,000 messages

Configuration	Sensitivity (95% CI)	Specificity (95% CI)	PPV (95% CI)	Alerts per 1,000 messages (95% CI; clinician review load)	Hazards caught per 1,000 messages (95% CI)	Hazards missed per 1,000 messages (95% CI)	Note
**Autonomous (physician-unassisted) triage benchmark**	≥ 0.800	≥ 0.800	—	—	—	≤ 16.5	Anchored to IDx-DR pivotal trial (sens 87.2%, spec 90.7%)
Best balanced single architecture (ActionHead (action recommender))	0.424 (0.351–0.501)	0.802 (0.783–0.820)	0.162 (0.130–0.199)	217 (199–235)	35.0 (27.8–44.0)	47.5 (39.0–57.7)	default threshold; baseline reference
Highest-sensitivity single architecture (Logistic regression + TF-IDF)	0.970 (0.931–0.987)	0.093 (0.080–0.107)	0.088 (0.076–0.102)	913 (899–924)	80.0 (68.9–92.7)	2.5 (1.1–5.8)	default threshold; reference for sens-floor analysis
Best balanced ensemble (soft_f1weighted)	0.582 (0.506–0.654)	0.581 (0.558–0.603)	0.111 (0.092–0.134)	433 (411–454)	48.0 (39.5–58.3)	34.5 (27.4–43.4)	hard- or soft-voting; reference for ensemble closing-the-gap finding
Best balanced cascade (ActionHead (action recommender) × Logistic regression + TF-IDF)	0.418 (0.346–0.494)	0.809 (0.791–0.827)	0.165 (0.132–0.203)	209 (192–228)	34.5 (27.4–43.4)	48.0 (39.5–58.3)	two-stage AND-rule; reference for cascade closing-the-gap finding
**Policy A**: high-recall screen (LogReg + TF-IDF@thresh)	0.855 (0.793–0.900)	0.282 (0.262–0.303)	0.097 (0.083–0.113)	729 (709–748)	70.5 (60.1–82.6)	12.0 (8.1–17.8)	sens > = 0.85 floor; all flagged messages sent to clinician confirmation queue
**Policy B**: disagreement-stratified triage (2 or more of 10 architectures flag, route to clinician review)	0.939 (0.892–0.967)	0.128 (0.113–0.144)	0.088 (0.076–0.102)	878 (863–892)	77.5 (66.6–90.0)	5.0 (2.7–9.2)	0–1 architectures flagging: autonomous benign; 2 or more: clinician review; 7 or more: autonomous escalation

Caption: Per-configuration sensitivity, specificity, positive predictive value, alert count per 1,000 messages (count of positive predictions), hazards caught per 1,000 messages (count of true positives), and hazards missed per 1,000 messages (count of false negatives) on the 2000-message real-world test set. All quantities are computed directly from empirical sensitivity and specificity on the held-out test set; the table reports counts of alerts and counts of misses rather than estimates of clinician time, full-time equivalents, or institutional staffing capacity, none of which were measured in the present study. The autonomous (physician-unassisted) triage benchmark row is included as the reference benchmark for hypothetical fully-autonomous deployment; the 165-hazard distribution would admit up to 16.5 missed hazards per 1,000 messages under the sensitivity ≥ 0.80 floor. Policy A and Policy B both produce residual miss rates below the symmetric-benchmark-implied maximum while producing alert volumes 2.53-fold (Policy A) and 3.03-fold (Policy B) higher than the best balanced single-architecture operating point (XGBoost with sentence-BERT embeddings at default threshold, 288 alerts per 1,000 messages)

## Discussion

Two principal findings emerge from this study. First, two operating points meeting the pre-specified sensitivity-floor target under clinician review of flagged messages were characterized for hazard detection in this Medicaid messaging population. A high-recall first-stage screen attains sensitivity 0.855 with 12.0 missed hazards per 1,000 messages and 729 alerts per 1,000. A disagreement-stratified clinician-review workflow attains sensitivity 0.939 with 5.0 missed hazards per 1,000 messages and 878 alerts per 1,000. Both configurations have residual miss rates below the 16.5 per 1,000 messages implied by a sensitivity floor of 0.80. The cost is alert volumes 2.5 to 3.0 times higher than the best balanced single-architecture operating point. The deployable pattern is the asymmetric-reward Conservative Q-Learning controller (or an equivalently threshold-tuned high-sensitivity classifier) serving as the first-stage screen, with the false-alarm volume routed to a clinician review queue rather than blocking workflow.

Second, no evaluated configuration attained the autonomous (physician-unassisted) triage benchmark (sensitivity 0.80 or higher and specificity 0.80 or higher simultaneously, without clinician review) for hazard detection on the real-world Medicaid messaging population. This held at every operating point on every receiver operating characteristic curve, across all 217 ensemble configurations, and across all 156 two-stage AND-rule cascade configurations. The result is a structural finding, not a threshold-calibration artifact. The best balanced single-architecture operating point was XGBoost with sentence-BERT at sensitivity 0.594 and specificity 0.592. The best balanced ensemble was the hard-voting three-of-13 rule at sensitivity 0.685 and specificity 0.575. Both fall substantially short of the autonomous (physician-unassisted) triage benchmark used here (sensitivity 0.80 or higher and specificity 0.80 or higher), anchored to the IDx-DR autonomous-diagnostic-AI pivotal trial [20]. The interpretation is that autonomous deployment of any evaluated architecture without clinician review is not supported by the present evidence on this population; clinician-reviewed deployment is required. This deployment pattern fits how primary care messaging already works [1, 3]. Medical assistants and care coordinators perform an initial triage and route messages to a physician whose inbox is already near capacity [4]. A high-sensitivity Stage 1 artificial intelligence screen would add to this human step rather than replace it, and is of operational relevance at safety-net practices, where the supply of primary care providers falls short of demand [5].

This finding is specific to the population evaluated. Messages in this Medicaid managed care patient-messaging service were written at substantially lower reading level than physician-scripted scenarios (mean grade 4.6 versus 5.8) and contained colloquialisms at substantially higher rates (59.1% versus 19.5%); abbreviation and implicit context rates were directionally lower in real-world messages than in physician scenarios. These linguistic differences, combined with the natural hazard-category mix of a Medicaid messaging service (described in Results), distinguish the patients evaluated here from the populations on whom prior clinical artificial intelligence evaluation has been conducted. The safety characteristics established for clinical artificial intelligence on academic medical center cohorts and physician-scripted scenarios are demonstrated here not to transfer to this population.

The harm asymmetry of clinical hazard detection (a missed hazard may cause irreversible patient harm, whereas a false alarm causes recoverable workflow burden) was reflected in the Conservative Q-Learning controller’s asymmetric reward design, with a 25-fold penalty on missed hazards relative to false alarms. This architectural commitment to sensitivity over specificity produces a natural first-stage screen in a cascaded workflow but does not, in itself, close the population gap. The architecture-level rank ordering, the absolute sensitivity attained, and the under-triage rates observed should be interpreted as the safety profile of these architectures on the population they are most often deployed to serve.

The 2026 multimodal Articulate Medical Intelligence Explorer demonstration [8] evaluated a frontier large language model on 105 scripted patient-actor scenarios across 32 non-multimodal and 9 multimodal qualitative axes rated by 18 specialists; the present study evaluates 2000 real Medicaid patient short message service messages against physician-adjudicated reference labels using quantitative receiver operating characteristic, F1, and Matthews correlation coefficient outcomes. The two paradigms are categorically distinct on three dimensions. The population dimension contrasts scripted patient actors at an academic medical center research site against real Medicaid patients across three U.S. states. The task dimension contrasts multi-turn multimodal diagnostic conversation against single-turn screening at message arrival triage. The outcome dimension contrasts specialist-rated qualitative axes against physician-adjudicated quantitative binary hazard detection. The two paradigms are complementary rather than competing. The multimodal Articulate Medical Intelligence Explorer characterizes the upper envelope of conversational diagnostic capability under controlled conditions. The present study characterizes the safety floor under operational deployment conditions on a population that the multimodal Articulate Medical Intelligence Explorer cohort does not represent. Liu and colleagues’ patient-portal-message triage work [12] is the closest published analog in task structure (artificial intelligence triage of patient-initiated text communication) but evaluated retrieval-augmented frontier large language models on academic medical center portal messages from a single quaternary-care institution, with patient and clinician populations whose linguistic patterns, reading level, and hazard category mix differ from the multi-state Medicaid short message service population evaluated here. The MedHELM benchmark suite [9] complements both by characterizing frontier-model performance on knowledge-intensive medical tasks; its findings on frontier-model competitiveness on knowledge tasks are not contradicted here, but the present results show that extending those findings to Medicaid messaging triage is non-trivial. Published clinical-message triage evaluations [32, 33] and decision-support comparisons in radiology [34] and oncology [35] have likewise informed the architectural design of the present study; the contribution of this work is to bring the same quantitative discipline to a multi-state Medicaid messaging population. A preprint on hallucination reduction in clinically safe large language models [36] is consistent with the present study’s finding that safety-augmented prompting affects the operating-point characteristics of frontier models but does not by itself match the performance of a controller trained on population-specific data.

The principal deployment implication of these findings is that current artificial intelligence architectures evaluated here are not deployable as autonomous single-stage screens for hazard detection in this multi-state Medicaid messaging population; deployment requires human-in-the-loop oversight at clinically meaningful rates. Under United States Food and Drug Administration Clinical Decision Support guidance, clinical decision support that recommends specific patient actions or is integrated with clinical workflow is subject to regulatory oversight; population-relevant performance evidence is a critical input to that oversight. The present findings constitute population-relevant performance evidence that does not support autonomous deployment of any single evaluated architecture, ensemble, or two-stage cascade for unsupervised hazard detection on this population.

The classical clinical-screening cascade (a high-sensitivity screen followed by a high-specificity confirmatory step) is a relevant framing for these results. In mammography, screening sensitivity prioritizes catching cancers and biopsy supplies confirmation. In human immunodeficiency virus testing, ELISA prioritizes sensitivity and Western blot supplies confirmation. In cervical cancer screening, Papanicolaou cytology prioritizes sensitivity and colposcopy with biopsy supplies confirmation. Policy A (a high-recall first-stage screen) instantiates this pattern for Medicaid messaging triage. The Stage 1 high-sensitivity artificial intelligence screen is the asymmetric-reward Conservative Q-Learning controller, or equivalently the threshold-tuned logistic regression with TF-IDF features at the sensitivity-floor-0.85 operating point. The Stage 2 high-specificity confirmatory step is clinician review of the screen-positive queue. In the present data, the clinician (rather than a second artificial intelligence) functioned as the higher-specificity confirmatory step. The intrinsic discriminative capacity of every evaluated automated Stage 2 on this population (frontier large language model, ensemble, or learned classifier) is too low to function as the confirmatory test without losing the screen-positive true positives that Stage 1 catches. For completeness, a two-artificial-intelligence cascade pairing the Stage 1 logistic regression screen with a Claude Opus 4.7 Stage 2 was evaluated and is reported in Additional file 1.

Policy A maps to the classical clinical-screening cascade in this setting. A Stage 1 artificial intelligence screen is sized for high sensitivity. All screen-positive messages route to a clinician confirmation queue, sized to the architecture’s empirical alert rate (729 alerts per 1,000 messages; Table S8). This pattern is consistent with prior art in clinical computer-aided detection, where artificial intelligence supplies pre-clinician filtering rather than autonomous decision-making. It is also the deployment pattern that the TRIPOD + AI [30] and DECIDE-AI [31] reporting guidelines anticipate for early-clinical-evaluation-stage decision support systems. The present results constitute the population-relevance evidence those frameworks require as a precondition for any deployment claim.

The longer-term deployment pathway, autonomous or semi-autonomous artificial intelligence hazard detection at the autonomous (physician-unassisted) triage benchmark on the Medicaid messaging population, will require closing the population gap documented here. The research agenda for closing the gap is outlined in future work below.

### What this study adds

- To the authors’ knowledge, this is the first published evaluation of clinical artificial intelligence safety on patient-initiated short message service communication from a multi-state Medicaid managed care population, with sample size, real-world linguistic profile, hazard-category mix, and physician-adjudicated reference labels all distinct from the academic medical center cohorts and physician-scripted scenarios on which prior frontier language model and clinical-decision-support evaluations have been conducted.
- No artificial intelligence tool or combination we tested was accurate enough to triage Medicaid patient messages on its own. This held for every single tool, all 217 ensembles, and all 156 two-stage cascades: none reached the pre-specified autonomous benchmark of sensitivity and specificity both 0.80 or higher (anchored on the IDx-DR autonomous-diagnostic-AI pivotal trial [20]). What did work was pairing a high-sensitivity tool with clinician review, the same screen-then-confirm pattern used in cancer and infectious-disease screening. Two configurations met the pre-specified sensitivity floor: a high-recall first-stage screen (sensitivity 0.855; 12.0 missed hazards and 729 alerts per 1,000 messages) and a workflow that routes any message flagged by two or more tools to a clinician (sensitivity 0.939; 5.0 missed hazards and 878 alerts per 1,000 messages). Both miss fewer hazards than the 0.80 sensitivity floor would allow, at a cost of 2.5 to 3.0 times more alerts than the best balanced single tool. The practical implication is that these tools belong in a human-in-the-loop workflow for this population, not in autonomous deployment. Reaching autonomous performance will require population-specific work: larger and better-labeled Medicaid messaging training data, a larger retrieval corpus, domain-adapted small models, active learning, and multi-turn evaluation, followed by prospective validation.
- Closing the gap from the best balanced operating point achievable here to the autonomous (physician-unassisted) triage benchmark motivates a structured research agenda anchored on larger population-relevant training corpora, expansion of the retrieval-augmented-generation corpus beyond the 1,280-example development set, domain-adapted small-model fine-tuning, active learning, and multi-turn evaluation, with prospective validation under DECIDE-AI Stage 2 as the gating downstream step.

### Development and deployment costs

The deployed decision support architecture and the supervised baselines incur development costs that the frontier language model configurations do not. Construction of the 1,280-example development corpus required three-physician adjudication of every message (hazard binary, hazard category, and triage action), with disagreement-resolution and senior-arbiter overhead. Training the Conservative Q-Learning controller, the constellation classifier, the ActionHead, and the supervised baselines required iterative hyperparameter selection on the validation split and Platt calibration. Periodic re-calibration is anticipated as the messaging population and clinical context evolve. The frontier language model configurations, in contrast, require no training data and no in-house compute, but introduce ongoing per-inference application-programming-interface cost, vendor dependency (model versioning, deprecation, terms-of-service change), and lower transparency into the model’s internal computation. The deployment-cost trade-off across these two cost structures is a function of message volume, expected horizon, regulatory documentation requirements, and institutional in-house engineering capacity, and is outside the scope of the present evaluation.

### Strengths and limitations

#### Strengths

A real-world test set drawn from a multi-state Medicaid managed care population (Virginia, Washington, Ohio) rather than a curated benchmark; physician-adjudicated reference labels on a per-message basis; a single deterministic pipeline run with SHA-256 input checksums producing every reported number end-to-end; per-message predictions retained and available on request for independent re-derivation of every table; pre-registered Hochberg-corrected paired comparisons across the 78-pair architecture matrix; Wilson 95% confidence intervals and 10,000-iteration bootstrap (seed = 42) for derived metrics; a configuration matrix spanning deployed clinical decision support, classical supervised baselines, and current-generation frontier large language models.

#### Limitations

This study has the following limitations, each named explicitly so the reader can weigh each against the reported findings.

##### Geographic scope

The participating multi-state clinically integrated network spans three U.S. states (Virginia, Washington, Ohio). Generalization to Medicaid populations in other states, to other payer populations, or to non-U.S. populations is not established and is identified as a future work priority.

##### Retrospective design

This is a retrospective evaluation. Prospective validation under a registered protocol is the appropriate DECIDE-AI Stage 2 next step and is identified as a future work priority.

##### Single-turn evaluation

Architectures were evaluated in single-turn screening at message arrival mode. The canonical test set carries per-message labels but no thread-level linkage; multi-turn dialogue evaluation will require Institutional Review Board amendment and a structured re-extraction of the messaging corpus with thread linkage and conversation-level ground truth, and is identified as a future work priority.

##### Message-level holdout with limited patient overlap

The train/test split was at the message level rather than the patient level. 41 of 1,679 unique test-set patients (2.4%) also appeared in the training set (with non-identical messages). This overlap is reported transparently; a patient-disjoint sensitivity analysis on the 1,638 patient-disjoint test-set patients is presented in the Additional file 1.

##### Message authorship

Messaging access is registered to the enrolled patient and identity verification is performed before each use, so messages are attributable to the enrolled patient’s record. For pediatric members (136 (8.1%) of patients) a parent or guardian composes the message on the member’s behalf. Because the linguistic features were computed from the message text, they characterize the input to each tool and do not depend on who composed a given message.

##### Generalizability

This is a care-management-engaged Medicaid subpopulation, not a representative sample of Medicaid enrollees. Relative to national Medicaid benchmarks it is substantially more adult, more heavily female, differently distributed by race/ethnicity, and far higher-utilizing, with roughly four times the national adult Medicaid emergency department visit rate (Table 3). These are the patients such a triage tool would serve in this setting, but the results should not be extrapolated to all Medicaid enrollees or to states outside Virginia, Washington, and Ohio.

##### Calibration and threshold selection

Thresholds and Platt probability calibration for every architecture with calibrated probability output were fit on a held-out validation split drawn from the development set and frozen before the single test pass; no thresholds, calibration constants, or hyperparameters were selected on the test set at any point. Test-set tuning would have produced optimistically biased operating-point estimates and is not present in any reported configuration. The visibly imperfect probability calibration on the real-world test set (Additional file 1, Figure S2) is itself a reported finding rather than a limitation to be corrected by recalibration: it reflects distributional shift between the validation set and the real-world Medicaid messaging population, and recalibration on the test set is a form of test-set tuning that is precluded under the present pre-registered protocol.

##### Frontier large language model coverage

Three frontier large language model providers (Anthropic Claude Opus 4.7, OpenAI GPT-5.5, and Google Gemini 3.1 Pro Preview) were evaluated under safety-augmented system prompts, and each was additionally evaluated with retrieval-augmented generation over the in-domain training corpus. The conclusions therefore concern current-generation frontier language model-class performance on this population, not vendor-specific claims. The safety-augmented prompt was the pre-specified single frontier-LLM configuration for the primary evaluation. A default-prompt comparator was not run on the held-out test set for any of the three providers, so a within-vendor safety vs. default prompt ablation is not available in this evaluation. Open-weight frontier-tier models and non-US frontier providers were also not evaluated.

##### Sample size

The 2000-message held-out test set was powered for primary architecture comparisons (see Sample size justification, Methods). The hazard-positive subgroup (165 hazards) supports the primary McNemar paired comparisons but limits within-category subgroup inference; subgroups with fewer than 30 hazard-positive cases are flagged with explicit small-sample warnings in equity-stratified and per-category analyses rather than suppressed.

##### Temporal validation

The held-out test set covers messages submitted during January 2023 through November 2025. The train/validation/test partition was performed at the message level rather than chronologically; messages from the training, validation, and test partitions are interleaved across the January 2023 through November 2025 interval. Random partitioning over a multi-year interval can in principle mask concept drift that would manifest under prospective deployment. Patient communication patterns and underlying disease prevalence may evolve, and temporal external validation on a forward-in-time messaging cohort is identified as a future work priority.

In summary, this study is a multi-state retrospective single-turn evaluation; the principal findings should be interpreted as population-relevance evidence for a use with clinician review of flagged messages cascade on Medicaid SMS triage, with prospective validation, multi-turn evaluation, geographic extension beyond the three participating states, and temporal external validation identified as the corresponding future work priorities.

### Future work: research agenda for closing the population gap

Closing the population gap documented in this study requires a structured research program. The gap is the distance from the best-balanced achievable operating point (sensitivity 0.685 and specificity 0.575 via the three-of-13 hard-voting ensemble) to the autonomous (physician-unassisted) triage benchmark (sensitivity 0.80 or higher and specificity 0.80 or higher). Five complementary directions are identified.

The first direction is expansion of the training corpus. The development set in this study comprised approximately 1,280 labeled examples. The hazard-positive subgroup within the development set is sparse, particularly for less-common categories such as obstetric and metabolic emergencies, and is a plausible source of the observed performance ceiling. Systematic labeling of additional Medicaid messaging samples, with hazard-category stratification, is the highest-priority research direction.

The second direction is an expansion of the retrieval corpus that supports retrieval-augmented generation. The present evaluation included retrieval-augmented generation over the 1,280-example labeled development set, which closed the gap on the physician-scripted comparison scenarios but not on the real-world Medicaid messaging test set. One interpretation is that the available retrieval corpus is too small and too narrowly distributed over the natural Medicaid messaging case mix to return informative neighbors for the colloquialism-rich, abbreviation-laden, implicit-context messages observed in deployment. Scaling the retrieval corpus to encompass a larger labeled set of in-domain population-relevant exemplars, with class stratification across the full 20 hazard categories, is the priority next step for retrieval-augmented generation in this population.

The third direction is domain-adapted small-model fine-tuning. Quantized low-rank adaptation of openly-available small large language models (such as Llama-3.1-8B or Mistral-7B) on the labeled Medicaid messaging corpus may improve discrimination over zero-shot frontier-model performance while preserving the operational advantages of locally-deployable inference.

The fourth direction is active learning on a held-out unlabeled corpus. Targeted labeling of the messages on which existing architectures disagree most strongly, drawn from the substantial unlabeled portion of the messaging stream, is an efficient route to closing the data gap without the cost of labeling messages on which architectures already agree.

The fifth direction is multi-turn evaluation. The present test set carries per-message labels rather than thread-level labels; multi-turn dialogue evaluation (in which the artificial intelligence can ask clarifying questions before recommending action) will require a structured re-extraction of the messaging corpus with thread linkage and conversation-level ground truth. Recent multi-turn conversational diagnostic artificial intelligence demonstrations on standardized patient actors [8] suggest that multi-turn capability may shift absolute performance for all architectures roughly in parallel; whether it specifically closes the population gap for low-literacy multilingual messaging is an open empirical question.

Two cross-cutting priorities complete the agenda. Temporal external validation will be required as patient communication patterns and the underlying disease prevalence evolve. External validation outside the three participating states (Virginia, Washington, Ohio) will be required before generalization to populations outside the participating multi-state clinically integrated network’s enrollees. Different payer populations, different age strata, additional geographies, and different primary languages will not necessarily reproduce the architecture-level rank ordering documented here. A prospective evaluation of clinical impact (whether deployment of the recommended cascade workflow in messaging triage reduces missed hazards relative to standard clinician-only review) is the logical final step once the foregoing have been completed.

## Conclusions

No artificial intelligence tool or combination we tested was accurate enough to triage Medicaid patient messages on its own. This held for every single tool, all 217 ensembles, and all 156 two-stage cascades: none reached the pre-specified autonomous benchmark of sensitivity and specificity both 0.80 or higher (anchored on the IDx-DR autonomous-diagnostic-AI pivotal trial [20]). What did work was pairing a high-sensitivity tool with clinician review, the same screen-then-confirm pattern used in cancer and infectious-disease screening. Two configurations met the pre-specified sensitivity floor: a high-recall first-stage screen (sensitivity 0.855; 12.0 missed hazards and 729 alerts per 1,000 messages) and a workflow that routes any message flagged by two or more tools to a clinician (sensitivity 0.939; 5.0 missed hazards and 878 alerts per 1,000 messages). Both miss fewer hazards than the 0.80 sensitivity floor would allow, at a cost of 2.5 to 3.0 times more alerts than the best balanced single tool. The practical implication is that these tools belong in a human-in-the-loop workflow for this population, not in autonomous deployment. Reaching autonomous performance will require population-specific work: larger and better-labeled Medicaid messaging training data, a larger retrieval corpus, domain-adapted small models, active learning, and multi-turn evaluation, followed by prospective validation.

## Supplementary Information

Below is the link to the electronic supplementary material.

Supplementary Material 1

## Data Availability

The complete analytic pipeline, including model training scripts, inference scripts, table and figure renderers, and audit utilities, is publicly available at https://github.com/sanjaybasu/rl-llm-safety. Per-architecture per-message predictions (architecture × message identifier × hazard probability × binarized prediction × ground-truth label × hazard category) are released as a supplementary file with the present manuscript. Raw patient message text is not publicly released because it remains under HIPAA data-use agreement; bona-fide researchers may request access from the corresponding author. The released supplementary file is sufficient for independent verification of receiver operating characteristic monotonicity, McNemar discordant-pair counts, paired bootstrap confidence intervals, and table-to-table arithmetic without access to the underlying message text.
